# Optogenetic calcium modulation in astrocytes enhances post-stroke recovery in chronic capsular infarct

**DOI:** 10.1126/sciadv.adn7577

**Published:** 2025-01-31

**Authors:** Jongwook Cho, Sangkyu Lee, Yeon Hee Kook, Jiyoung Park, Won Do Heo, C. Justin Lee, Hyoung-Ihl Kim

**Affiliations:** ^1^ Department of Biomedical Science and Engineering, Gwangju Institute of Science and Technology (GIST), Gwangju 61005, Republic of Korea.; ^2^ Center for Cognition and Sociality, Institute for Basic Science, Daejeon 34126, Republic of Korea.; ^3^ Department of Biological Sciences, Korea Advanced Institute of Science and Technology (KAIST), Daejeon 34141, Republic of Korea.; ^4^ Department of Neurosurgery, Presbyterian Medical Center, 365 Seowon-ro, Wansan-gu, Jeonju-si, Jeollabuk-do 54987, Republic of Korea.

## Abstract

Stroke is caused by disruption of cerebral blood flow, leading to neuronal death and dysfunction in the interconnected areas, which results in a wide range of severe symptoms depending on the specific brain regions affected. While previous studies have primarily focused on direct modulation of neuronal activity for post-stroke treatment, accumulating evidence suggests that astrocytes may play a critical role in post-stroke progression and could serve as a potential therapeutic target for recovery. In this study, we investigate the effects of selective modulation of astrocytic calcium signals on chronic stroke using OptoSTIM1, an optogenetic tool that activates endogenous calcium channels. In contrast to channelrhodopsin-2 (ChR2), OptoSTIM1 robustly elevates astrocytic calcium levels, sustaining the increase for over 10 min upon a single activation. The calcium elevation in astrocytes in the ipsilesional sensory-parietal cortex leads to remarkable recovery from post-stroke impairment. Thus, manipulating intracellular calcium levels in astrocytes holds promise as a potential therapeutic strategy for improving recovery following a stroke.

## Teaser

The modulation of astrocytic calcium signals promotes functional recovery in chronic stroke model.

## INTRODUCTION

Stroke is caused by a lack of blood flow in brain tissue and is a catastrophic event that leads to long-term disability in adults. It profoundly affects their quality of life and imposes a substantial economic burden on nationwide management (*1*). Especially, subcortical stroke accounts for approximately 30% of all ischemic strokes in humans and is associated with an unfavorable prognosis. Although the precise cellular and molecular mechanisms underlying post-stroke disability remain unclear, it is widely recognized that various neuromodulation strategies, such as magnetic or electrical stimulation, may offer benefits in enhancing stroke recovery, even in chronic cases. These strategies aim to increase neuronal excitability and promote neuroplasticity, thus facilitating the restoration of neuronal connectivity (*2*, *3*). While previous studies have primarily focused on neurons as the primary targets for post-stroke recovery (*4*, *5*), accumulating evidence suggests that glial cells also play critical roles in both the development of disability and the recovery process in chronic stroke (*6*, *7*).

Astrocytes, one of the most abundant cell types in the central nervous system, actively participate in a wide range of physiological and pathological processes in the brain that extend beyond their traditional role in “housekeeping functions,” including maintenance of the blood-brain barrier and the clearance of metabolic wastes and neurotransmitters (*8*). By receiving extracellular signals, various receptors and channels in astrocytes trigger distinct patterns of intracellular calcium signals in both the soma and processes (*9*). These calcium signals, in turn, regulate numerous cellular processes, including the release of gliotransmitters [e.g., glutamate, d-serine, adenosine 5′-triphosphate (ATP), and γ-aminobutyric acid (GABA)] and gene expression (*10*–*12*). Astrocytes establish close spatial relationships with neuronal compartments, enabling them to finely modulate neuronal activity and synaptic plasticity (*13*–*15*). Thus, it is increasingly evident that astrocytes, not just neurons, play a crucial role in information processing in the brain and directly contribute to diverse brain functions in both healthy and diseased states.

In the past decade, advancements in optogenetics have provided unprecedented means to precisely modulate target biological processes in both space and time through light stimulation. Opsin-based optogenetic approaches, exemplified by channelrhodopsin2 (ChR2), enable light-gated ion flow across the plasma membrane in response to light, which modulates membrane potential (*16*). These tools allow bidirectional control over the excitable cells, including neurons and muscle cells (*17*, *18*). While ChR2 has been used in several studies to modulate calcium signals in astrocytes, which are nonexcitable cells in the brain, serious concerns arise regarding the specificity of this approach in targeting calcium modulation. This is because ChR2 and its variants are nonselective cation channels that allow entry of not only Ca^2+^ but also Na^+^, K^+^, and H^+^ (*19*, *20*). Moreover, a recent study reported that light stimulation of ChR2-expressing astrocytes leads to extracellular K^+^ elevations, further complicating the interpretation of experimental findings (*21*).

Previously, we introduced an optogenetic technique called OptoSTIM1, which allows for the activation of calcium channels by inducing light-dependent oligomerization of the STIM1 protein (*22*). Unlike opsin-based optogenetic tools, OptoSTIM1 specifically targets endogenously expressed CRAC (calcium release–activated calcium) channels, which are highly selective for calcium ions and ubiquitously expressed in both excitable and nonexcitable cells. Through the use of OptoSTIM1, we were able to achieve spatiotemporal modulation of calcium signals in various cell types and control specific brain functions in vivo. In this study, we examine the hypothesis that selective modulation of astrocytic calcium using OptoSTIM1 can induce changes in the brain state in a chronic stroke model by modulating the communication between astrocytes and neurons, thereby contributing to post-stroke recovery.

## RESULTS

### Optogenetic modulation of calcium signals in astrocytes

**Fig. 1.**
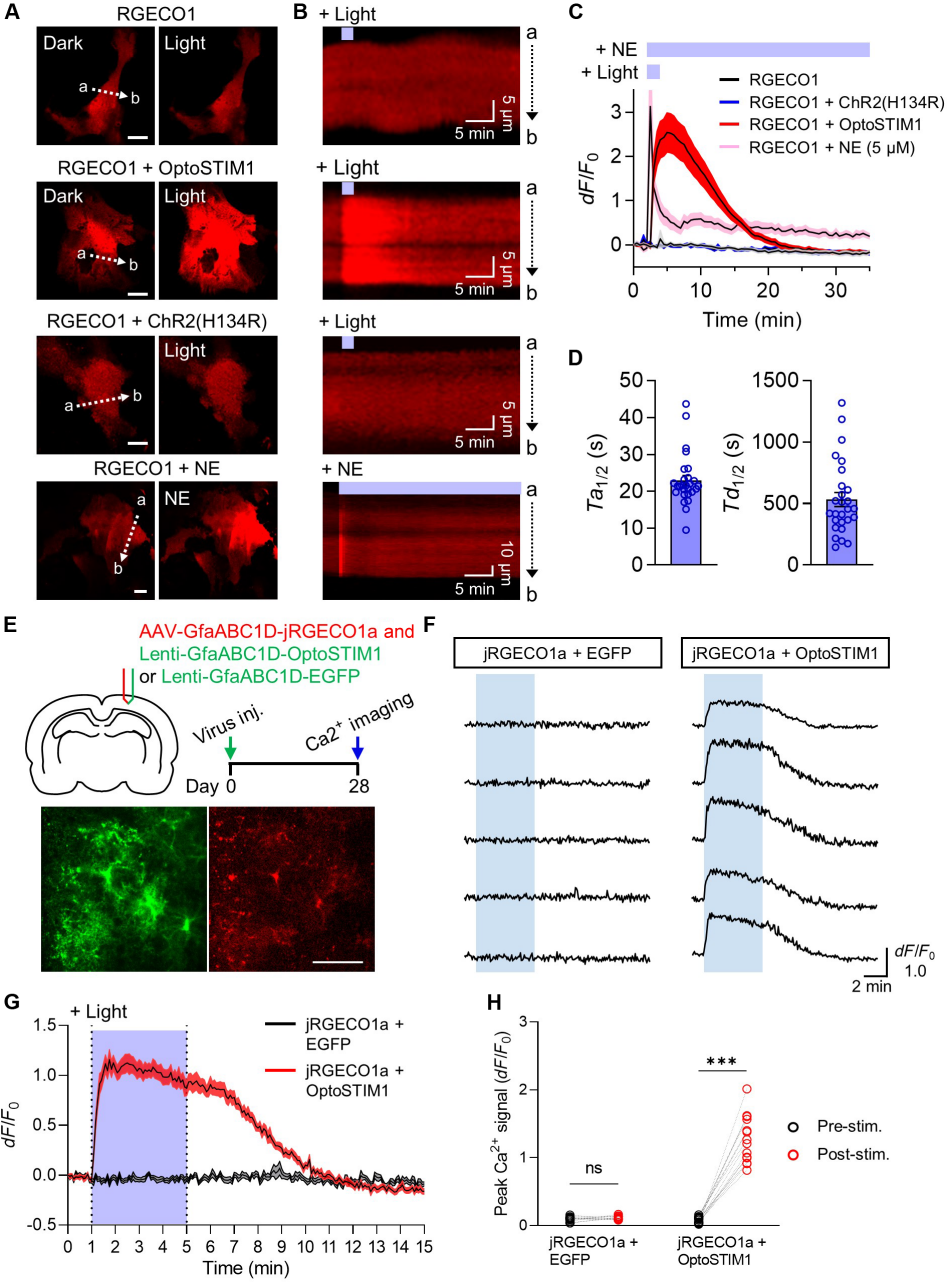
Light-induced Ca^2+^ influx in astrocytes through OptoSTIM1 activation. (**A**) Fluorescence images of cultured astrocytes expressing RGECO1 along with OptoSTIM1 or ChR2(H134R). Scale bars, 20 μm. (**B**) Kymographs corresponding to the white dotted lines in (A), showing RGECO1 fluorescence upon light stimulation. (**C**) A graph illustrating changes in RGECO1 intensity upon light stimulation or NE (5 μM) treatment. (**D**) Graphs showing the time required to reach half-maximal intensity (left, *Ta*_1/2_, “a” refers to activation) and half-basal intensity from the maximal intensity of RGECO1 fluorescence (right, *Td*_1/2_, where “d” refers to deactivation) upon OptoSTIM1 activation. (**E**) Schematic diagram and experimental timeline of the virus injection and two-photon Ca^2+^ imaging. Representative two-photon images of a brain slice demonstrate the viral expression of Lenti-GfaABC1D-OptoSTIM1 (green) and AAV-GfaABC1D-jRGECO1a (red) in the SPC. Scale bar, 100 μm. (**F**) Representative Ca^2+^ traces of RGECO1 intensity upon light stimulation. (**G**) A graph illustrating changes in the average fluorescent ratio for the GfaABC1D-EGFP and GfaABC1D-OptoSTIM1 groups. The purple-shaded area indicates the period of light stimulation. (**H**) Comparison of peak Ca^2+^ signals in the GfaABC1D-EGFP and GfaABC1D-OptoSTIM1 groups before and after light stimulation [repeated-measures two-way analysis of variance (ANOVA) with Sidak’s multiple comparisons, *F*_1,24_ = 130.6, *P* < 0.0001]. Error bars represent means ± SEM. ***P < 0.001; ns, nonsignificant.

To examine the effectiveness of OptoSTIM1 in inducing light-dependent calcium influx in astrocytes, we expressed OptoSTIM1 or ChR2(H134R) along with R-GECO1, a red fluorescent calcium indicator, in cultured astrocytes. Upon transient illumination (1 Hz) with blue light for 2 min at 30-s intervals, we observed a remarkable and reversible increase in R-GECO1 fluorescence in astrocytes expressing OptoSTIM1, while ChR2-expressed astrocytes did not elevate R-GECO1 fluorescence (Fig. 1, A to C). Notably, there was no discernible difference in basal calcium dynamics between astrocytes expressing OptoSTIM1 and those without expression (fig. S1A and movie S1). Treatment of astrocytes with norepinephrine (NE), a ligand for activating endogenous adrenergic receptors, exhibited a similar maximal fold change in calcium increase to that observed with OptoSTIM1 activation, suggesting that the calcium levels induced by OptoSTIM1 are within a physiologically relevant range. Notably, unlike the calcium dynamics in the OptoSTIM1-activating cells, calcium levels in NE-treated cells rapidly decreased, maintaining a modest level for over 30 min. The lack of calcium increase observed with ChR2 activation in our experiment contrasts with previous studies (*23*, *24*). As this discrepancy might be attributed to the light-stimulating conditions, we activated astrocytes expressing ChR2 with a higher frequency (16 Hz) and observed a noticeable elevation in calcium levels. However, even in this condition, ChR2-expressing cells elicited calcium signals with lower amplitudes and shorter durations than those in the OptoSTIM1 group (fig. S1, B and C, and movie S2). The activation and deactivation kinetics (*Ta*_1/2_, *Td*_1/2_) of OptoSTIM1 were 22.9 ± 1.35 s and 532.7 ± 57.84 s (means ± SEM), respectively (Fig. 1D).

Next, we conducted two-photon calcium imaging in brain slices to monitor the calcium influx in astrocytes of the sensory-parietal cortex (SPC) upon activation of OptoSTIM1. We injected a mixture of viruses into the SPC to express jRGECO1a along with OptoSTIM1 or EGFP in astrocytes (Fig. 1E). Fluorescence images of SPC astrocytes coexpressing jRGECO1a and OptoSTIM1 were captured with 5-s intervals for 15 min. We observed an immediate increase in intracellular calcium levels in astrocytes expressing OptoSTIM1 upon blue light stimulation, whereas astrocytes expressing (EGFP) did not exhibit any noticeable change in calcium levels (Fig. 1, F to H, and movie S3). Therefore, these results indicate that activation of OptoSTIM1 effectively elevates intracellular calcium levels in astrocytes.

### Optogenetic modulation of calcium signals in astrocytes of SPC promotes post-stroke recovery

**Fig. 2.**
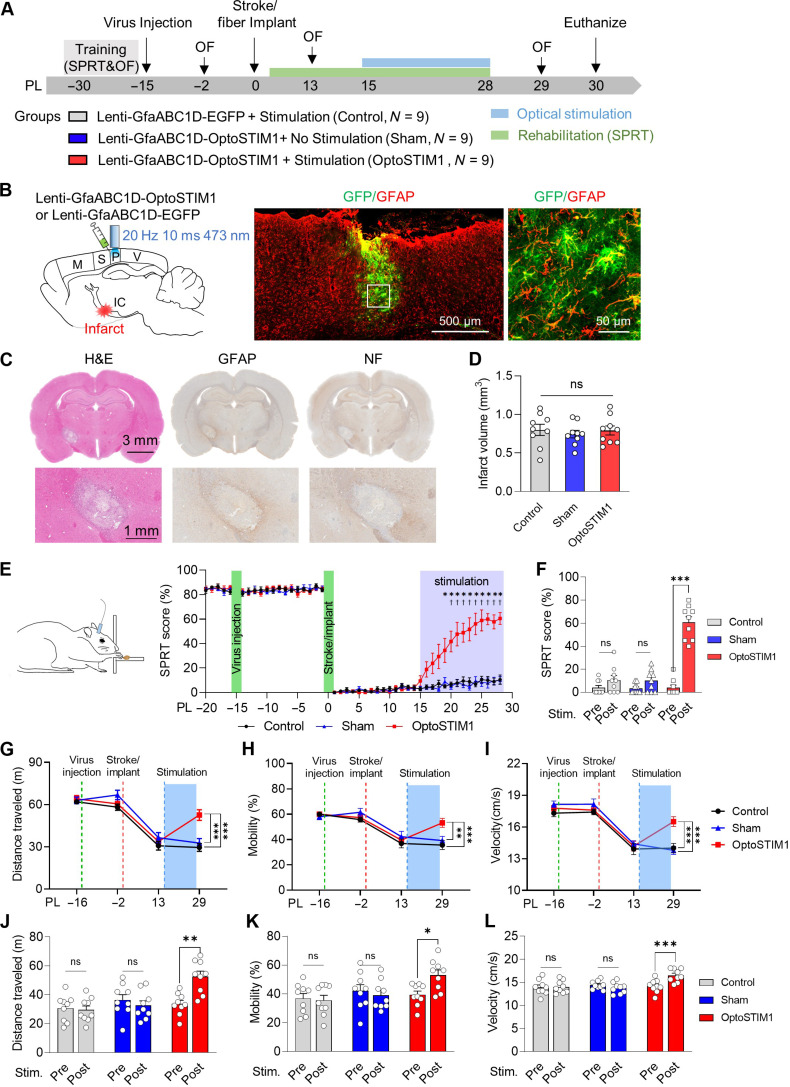
OptoSTIM1 activation in SPC astrocytes leads to motor recovery in a chronic capsular infarct model. (**A**) Experimental design and timeline of surgeries and behavioral tests. SPRT, single pellet reaching task; OF, open field test. (**B**) Schematic diagram of the capsular infarct model and viral injection in the SPC. M, motor cortex; S, sensory cortex; P, parietal cortex; V, visual cortex. (**C**) Capsular infarcts stained for hematoxylin and eosin (H&E) (left), GFAP (center), and neurofilament (right). (**D**) Volume of capsular infarcts in three different groups (one-way ANOVA with Tukey’s multiple comparisons, *F*_2,24_ = 0.2391, *P* = 0.7892). (**E**) Schematic diagram and daily performance of SPRT (repeated-measures two-way ANOVA with Geisser-Greenhouse correction and Tukey’s multiple comparisons, *F*_92,1104_ = 14.56, *P* < 0.0001; *OptoSTIM1 versus Control; †OptoSTIM1 versus Sham). The purple-shaded area indicates the period of optical stimulation. (**F**) Changes in SPRT performance after optogenetic stimulation (repeated-measures two-way ANOVA with Sidak’s multiple comparisons, *F*_2,24_ = 72, *P* < 0.0001). (**G** to **I**) Changes in locomotor activity in the open field arena, showing distance traveled (m), mobility (%), and locomotor velocity (cm/s) [repeated-measures two-way ANOVA with Tukey’s multiple comparisons (G) *F*_6,72_ = 5.448, *P* = 0.0001; (H) *F*_6,72_ = 2.852, *P* = 0.0151; (I) *F*_6,72_ = 4.673, *P* = 0.0005]. (**J** to **L**) Changes in distance traveled (m), mobility (%), and locomotor velocity (cm/s) before and after optogenetic stimulation [repeated-measures two-way ANOVA with Sidak’s multiple comparisons, (J) *F*_2,24_ = 7.456, *P* = 0.0030; (K) *F*_2,24_ = 3.504, *P* = 0.0462; (L) *F*_2,24_ = 8.626, *P* = 0.0015]. Error bars represent means ± SEM. *P < 0.05, **P < 0.01, and ***P < 0.001.

Given that task-specific exercise combined with SPC stimulation enhances behavioral recovery in a chronic capsular infarct model (*25*, *26*), we investigated the potential of alleviating post-stroke disability through optogenetic calcium modulation in astrocytes of the SPC region. After selectively expressing OptoSTIM1 in astrocytes of the ipsilesional SPC of rats through viral transduction, the animals were divided into OptoSTIM1-stimulation group (OptoSTIM1, *N* = 9), which underwent optical stimulation, and sham-operated group (Sham, *N* = 9), which did not receive optical stimulation. In addition, a control group (Control, *N* = 9) received a control virus (GfaABC1D-EGFP) in the same area and optical stimulation. Subsequently, we conducted photothrombotic stroke lesioning in the posterior limb of the internal capsule (PLIC) 4 weeks after virus injection (Fig. 2, A to C). We confirmed that the expressed proteins were predominantly localized in astrocytes. The volume of virus transduction was 0.30 ± 0.05 mm^3^ in the EGFP group and 0.16 ± 0.02 mm^3^ (means ± SEM) in the OptoSTIM1 group. Specificity was 92.01% in the EGFP group and 95.19% in the OptoSTIM1 group (Fig. 2B and fig. S2). Animals in all experimental groups exhibited similar infarct volumes (Fig. 2D). Following confirmation of persistent motor impairment for at least 2 weeks, we activated OptoSTIM1 for 1 hour using blue laser stimulation (20 Hz, 10 ms, 1.5 mW) and performed a daily single pellet reaching task (SPRT) to assess fine motor skill in the contralesional forelimb. The group of animals subjected to OptoSTIM1 activation showed a remarkable improvement in reaching performance compared to the control and sham groups after days of light stimulation, eventually reaching 60.6% of the SPRT score (Fig. 2, E and F). In the open field test, which measures general locomotor activity, we observed a significant increase in traveled distance, mobility, and velocity in the group with OptoSTIM1 activation in the SPC astrocytes, while the control and the sham-operated groups did not show significant improvements in locomotor activity (Fig. 2, G to L). Notably, the activation of ChR2 expressed in astrocytes using the same illumination protocol did not improve post-stroke motor impairments (fig. S3). Together, these results demonstrate that astrocyte calcium modulation using OptoSTIM1 effectively reduces post-stroke deficits, providing support for the critical role of astrocytes in this process.

### Optogenetic calcium modulation in SPC astrocytes reduces the diaschisis and restores corticocortical circuits

**Fig. 3.**
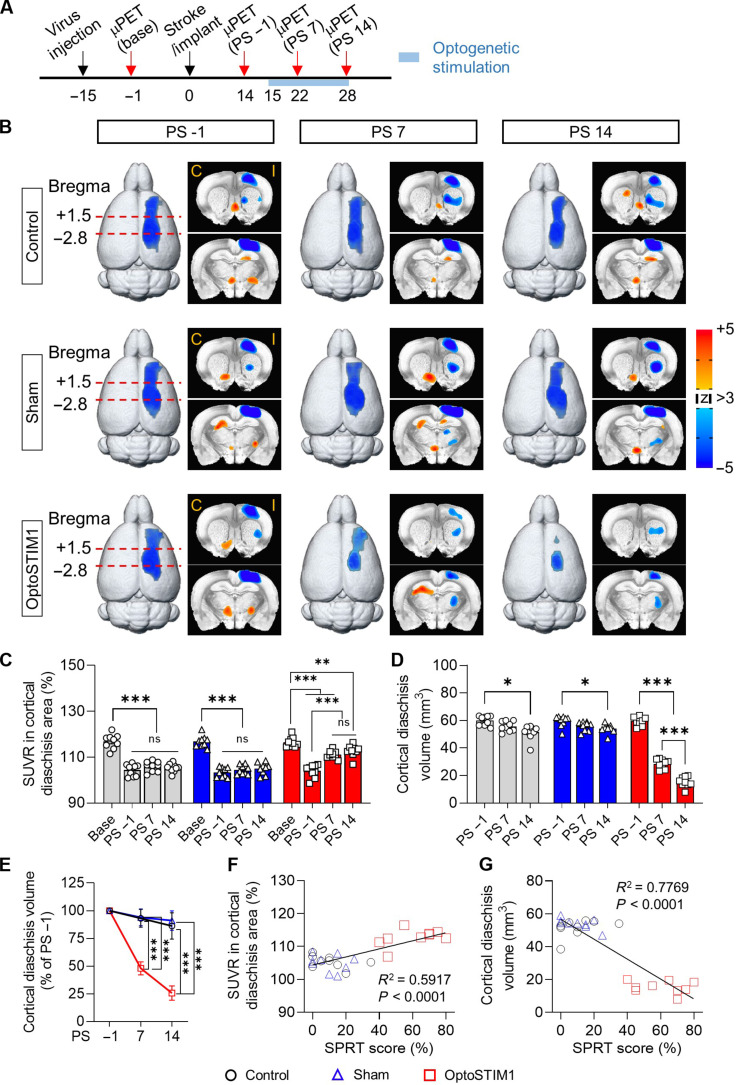
OptoSTIM1 stimulation in the SPC reverses cortical diaschisis in a chronic stroke model. (**A**) Experimental timeline of ^18^F-FDG-microPET study. PS, post-stimulation. (**B**) Longitudinal FDG-micoPET imaging showing time-dependent changes in cortical diaschisis after capsular infarct lesioning (3dLME in AFNI, *P* = 0.001, FDR of *q* < 0.05). The image analysis was performed between pre-lesional (baseline) and post-lesional images (PS −1, PS 7, and PS 14) to identify longitudinal changes in cortical diaschisis and regional glucose metabolism following astrocytic optogenetic stimulation. The color scale bar represents *z* scores, where positive values (orange-red) indicate regions with increased glucose metabolism relative to baseline, and negative values (blue) indicate regions with decreased glucose metabolism. C, contralesional; I, ipsilesional. (**C**) Changes in SUVR in the cortical diaschisis area (repeated-measures two-way ANOVA with Geisser-Greenhouse correction and Tukey’s multiple comparisons, *F*_6,72_ = 13.84, *P* < 0.0001). (**D**) Time-dependent changes in cortical diaschisis volume (repeated-measures two-way ANOVA with Geisser-Greenhouse correction and Tukey’s multiple comparisons, *F*_4,48_ = 81.53, *P* < 0.0001). (**E**) Time-dependent changes in the normalized cortical diaschisis volume (repeated-measures two-way ANOVA with Geisser-Greenhouse correction and Tukey’s multiple comparisons, *F*_4,48_ = 93.74, *P* < 0.0001). (**F**) Positive correlation between SUVR in cortical diaschisis area and SPRT scores (linear regression, *F*_1,25_ = 36.23, *P* < 0.0001). (**G**) Negative correlation between the volume of cortical diaschisis and SPRT scores (linear regression, *F*_1,25_ = 87.04, *P* < 0.0001). Error bars represent means ± SEM. *P < 0.05, **P < 0.01, and ***P < 0.001.

After a stroke, glucose hypometabolism in brain regions distant from the focal lesion has been frequently observed (*27*, *28*). These changes, referred to as diaschisis, are closely associated with clinical symptoms and stroke recovery (*29*, *30*). To investigate cortical diaschisis changes and examine the impact of optogenetic calcium modulation in astrocytes, we performed longitudinal 2-deoxy-2-[18F]-fluoro-d-glucose micro-positron emission tomography (FDG-microPET) imaging. Four scans were conducted on all animals: a baseline scan after viral injection but before stroke lesioning (base), a scan after stroke induction before optical stimulation (PS −1), and two scans at 7 and 14 days following the onset of stimulation (PS 7 and PS 14, respectively) (Fig. 3A). To visualize the diaschisis, FDG-microPET images were analyzed by comparing pre-lesional and post-lesional longitudinal scans. We quantified glucose metabolic activity in a specific brain area using the standardized uptake value ratio (SUVR), calculated by normalizing the SUV of region of interest (ROI) to the mean SUV of the entire brain. Cortical diaschisis was defined using statistical thresholding with maps corrected and thresholded at a significance level of *P* < 0.001 and a false discovery rate (FDR) of *q* < 0.05. In practice, the diaschisis area showed a decrease in SUVR of at least 10% compared to the pre-lesion baseline.

All groups exhibited extensive glucose hypometabolism in the ipsilesional motor and sensory cortex following capsular infarction (Fig. 3B and fig. S4). The control and sham-operated groups showed an initial reduction in SUVR in the cortical diaschisis area, with no significant changes observed throughout the stimulation period. However, the OptoSTIM1 group exhibited a significant increase in SUVR by PS 14 (Fig. 3C). In addition, we observed a significant reduction in cortical diaschisis volume from PS 7 onwards upon OptoSTIM1 activation, whereas the control and sham-operated groups showed a slight decrease in diaschisis volume only at PS 14 (Fig. 3, D and E). We observed significant positive correlations between SPRT scores and SUVR and negative correlations between SPRT scores and diaschisis volume (Fig. 3, F and G, and fig. S5). Given that all groups exhibited diaschisis across multiple cortical areas following capsular infarction and that OptoSTIM1 stimulation in the SPC led to increased metabolic activity (SUVR) in these regions and behavioral recovery, this suggests the functional engagement of the “corticocortical circuit.” In addition, these results indicate that the severity (i.e., extent and magnitude) of glucose hypometabolism is closely associated with stroke-related behavioral outcomes, and modulation of astrocyte calcium can reverse glucose hypometabolism, leading to functional recovery in the capsular infarct model.

**Fig. 4.**
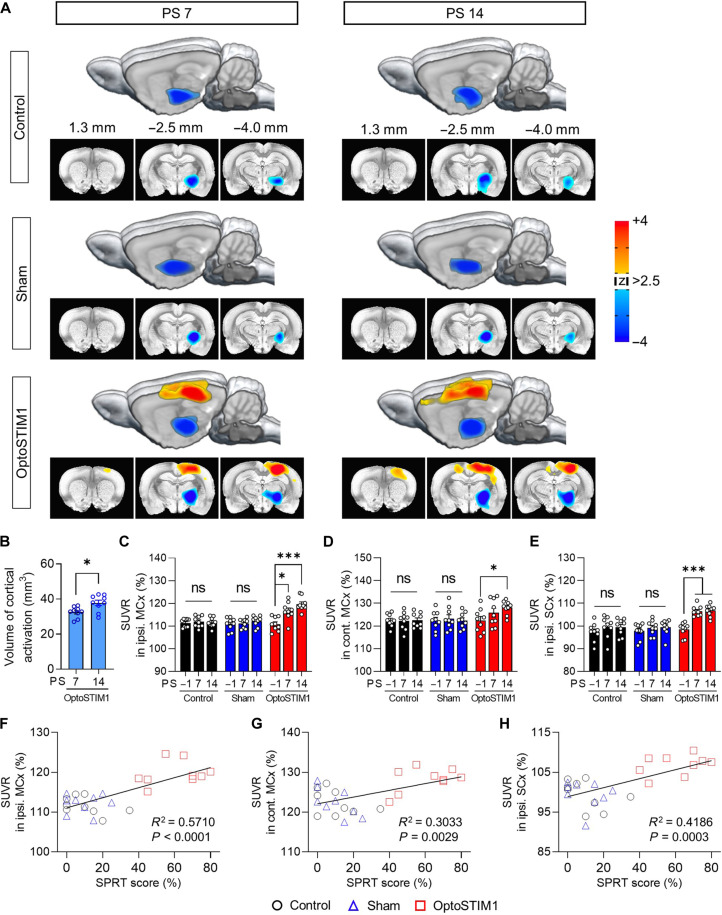
OptoSTIM1 stimulation in the SPC restores corticocortical circuits in a chronic stroke model. (**A**) 3D-rendered FDG-microPET images illustrating the activation of corticocortical circuits following OptoSTIM1 astrocytic stimulation (3dClustSim in AFNI *P* = 0.01, α = 0.05, *k* < 39). The color scale bar represents *z* scores, where positive values (orange-red) indicate regions with increased glucose metabolism relative to baseline, and negative values (blue) indicate regions with decreased glucose metabolism. (**B**) Changes in volume of cortical activation in the OptoSTIM1 group (paired *t* test, *t* = 2.963, *P* = 0.0181). (**C** to **E**) Changes in SUVR in the ipsilateral motor cortex, contralateral motor cortex, and ipsilateral sensory cortex, respectively [repeated-measures two-way ANOVA with Geisser-Greenhouse correction and Tukey’s multiple comparisons, (C) *F*_4,48_ = 8.760, *P* < 0.0001; (D) *F*_4,48_ = 2.662, *P* = 0.0437; (E) *F*_4,48_ = 15.47, *P* < 0.0001]. (**F** to **H**) Positive correlation between SPRT score and SUVR in the ipsilateral motor cortex, contralateral motor cortex, and ipsilateral sensory cortex, respectively [linear regression, (F) *F*_1,25_ = 33.28, *P* < 0.0001; (G) *F*_1,25_ = 10.88, *P* = 0.0029; (H) *F*_1,25_ = 18.00, *P* = 0.0003]. Error bars represent means ± SEM. *P < 0.05 and ***P < 0.001.

To further investigate the effect of optogenetic modulation of SPC astrocytes, we analyzed FDG-microPET images acquired before and after stimulation. At PS 7, OptoSTIM1 activation resulted in significant cortical activation in the ipsilesional motor and sensory cortex, indicating the activation of corticocortical circuits (Fig. 4A). At PS 14, cortical excitability was even more widespread, extending to both the ipsilesional and contralesional motor cortex (Fig. 4, A and B). No changes were observed in the control and sham groups. Notably, we observed a significant increase in SUVR in the bilateral motor cortex, particularly in the ipsilesional sensory cortex (i.e., stimulation area), under OptoSTIM1 activation. In contrast, the other groups did not exhibit any increase in SUVR (Fig. 4, C to E). We also found a significant positive correlation between SUVR in the bilateral motor cortex and the ipsilateral sensory cortex and the SPRT score (Fig. 4, F to H, and fig. S6). Furthermore, we conducted an additional microPET study to assess the effect of OptoSTIM1 stimulation in nonstroke rats, thus excluding the influence of stroke modeling. We observed a remarkable increase in regional glucose metabolism upon OptoSTIM1 activation in the target area (fig. S7). Together, these findings suggest that the activation of OptoSTIM1 is likely to drive astrocytic activation, which could enhance neuronal activity and synaptic transmission via calcium-mediated release of gliotransmitters (*12*, *31*–*33*), potentially contributing to functional recovery following a stroke.

### Astrocytic calcium modulation after a stroke increases neuronal activity and BDNF expression

**Fig. 5.**
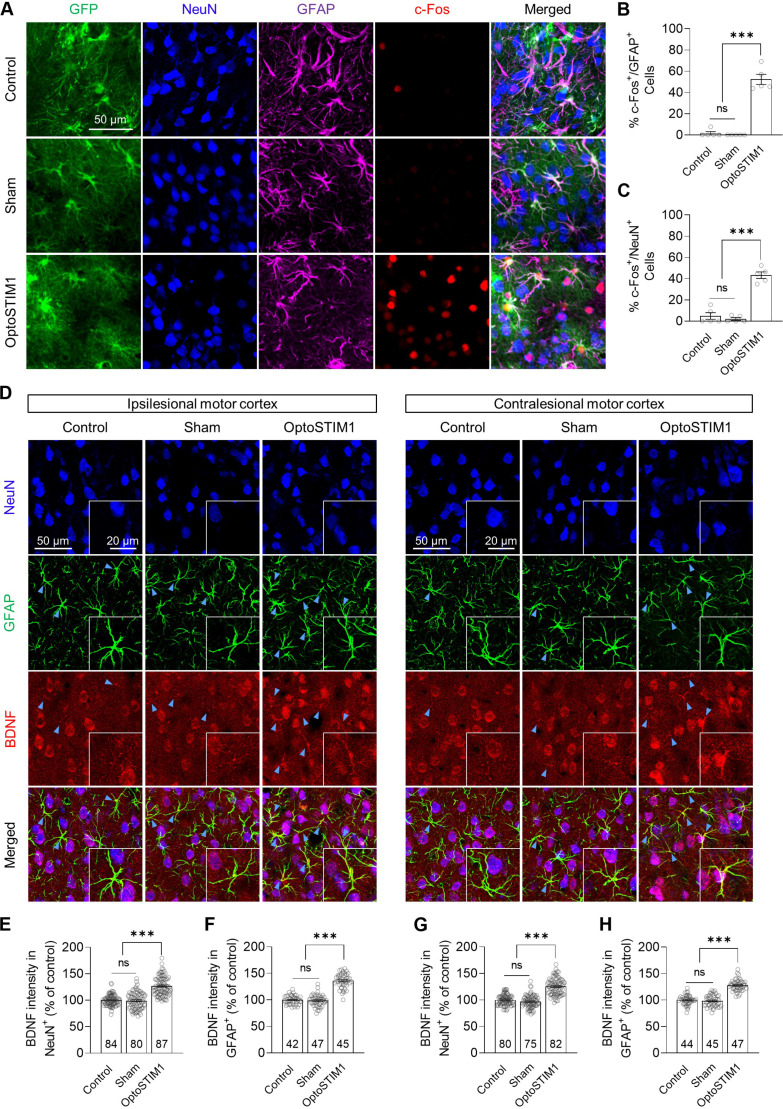
Astrocytic calcium modulation using OptoSTIM1 increases neuronal activity and BDNF expression. (**A**) Representative confocal images showing the colocalization of GFP, NeuN, GFAP, and c-Fos in the SPC. (**B**) Quantification of the population of c-Fos–positive cells in astrocytes in the virus-expressing area (one-way ANOVA with Tukey’s multiple comparisons, *F*_2,12_ = 103.3, *P* < 0.0001). (**C**) Quantification of the population of c-Fos–positive cells in neurons in the area adjacent to the virus-expressing region (one-way ANOVA with Tukey’s multiple comparisons, *F*_2,12_ = 76, *P* < 0.0001). (**D**) Representative confocal images showing staining of NeuN, GFAP, and BDNF in the bilateral motor cortex. (**E**) Quantification of BDNF intensity in NeuN-positive neurons in the ipsilateral motor cortex (one-way ANOVA with Tukey’s multiple comparisons, *F*_2,248_ = 94.75, *P* < 0.0001). (**F**) Quantification of BDNF intensity in GFAP-positive astrocytes in the ipsilateral motor cortex (one-way ANOVA with Tukey’s multiple comparisons, *F*_2,131_ = 165.7, *P* < 0.0001). (**G**) Quantification of BDNF intensity in NeuN-positive neurons in the contralateral motor cortex (one-way ANOVA with Tukey’s multiple comparisons, *F*_2,234_ = 122.6, *P* < 0.0001). (**H**) Quantification of BDNF intensity in GFAP-positive astrocytes in the contralateral motor cortex (one-way ANOVA with Tukey’s multiple comparisons, *F*_2,133_ = 123, *P* < 0.0001). The number on each bar refers to the number of cells analyzed. BDNF-positive astrocytes are indicated by blue arrows. Error bars represent means ± SEM. ***P < 0.001.

To explore the molecular mechanisms underlying post-stroke recovery through astrocyte calcium modulation, we assessed the expression of c-Fos, an immediate early gene. Animals in the control and OptoSTIM1 groups were exposed to 30 min of light stimulation (473 nm, 20 Hz, 10 ms, 1.5 mW), while the sham group did not receive blue light exposure. OptoSTIM1 activation resulted in a significant increase in c-Fos expression not only in OptoSTIM1-positive astrocytes but also in neighboring OptoSTIM1-negative neurons upon light stimulation (Fig. 5, A to C). Specifically, 52.3% of OptoSTIM1-positive astrocytes were c-Fos positive (Fig. 5B), and 43.3% of adjacent neurons exhibited elevated c-Fos expression (Fig. 5C). This finding suggests that elevating intracellular calcium levels in astrocytes using OptoSTIM1 not only modulates astrocyte function but also affects nearby neuronal activity.

On the basis of our observation of restored corticocortical circuitry with OptoSTIM1 activation (Fig. 4), we further investigated whether OptoSTIM1 activation in astrocytes enhances the expression of brain-derived neurotrophic factor (BDNF), a secreted neurotrophin crucial for activity-dependent synaptic plasticity. Analysis of BDNF expression in the bilateral motor and ipsilesional sensory cortex, a brain region critical for post-stroke recovery (*34*), revealed a significant increase in BDNF expression in both astrocytes and neurons in the OptoSTIM1 group, suggesting that enhanced synaptic plasticity mediated by astrocyte calcium modulation may play a key role in post-stroke recovery (Fig. 5, D to H, and fig. S8).

## DISCUSSION

In this study, we demonstrate the effects of selective modulation of astrocytic calcium signals in a chronic stroke model using OptoSTIM1, a unique optogenetic tool that allows specific control of intracellular calcium levels through activation of endogenous CRAC channels. We demonstrated that optogenetic modulation of astrocyte calcium can enhance functional recovery in a rat model of chronic stroke. Following the stroke, the activation of OptoSTIM1 in SPC astrocytes led to improvements in fine motor skills of the impaired forelimb and general locomotor activities. In addition, FDG-microPET imaging revealed that OptoSTIM1 activation in astrocytes facilitated the restoration of the corticocortical circuit with a remarkable reduction in cortical diaschisis volume, which is a critical process contributing to post-stroke recovery in the capsular stroke model (*26*, *35*). Our study also showed that OptoSTIM1 activation resulted in a significant increase in c-Fos expression not only in OptoSTIM1-positive astrocytes but also in OptoSTIM1-negative neurons, highlighting the close astrocyte-neuronal interaction. Analysis of plasticity marker BDNF showed a significant increase in BDNF expression in both astrocytes and neurons following OptoSTIM1 stimulation, implying enhanced synaptic plasticity through astrocyte calcium channel modulation. This study thus underscores the effectiveness of astrocytic modulation through the activation of endogenous calcium channels with OptoSTIM1 for augmenting post-stroke recovery in capsular infarcts.

Now, various cortical neuromodulation strategies have been explored (*36*, *37*). However, cortical neuromodulation affects all types of cells located in the stimulation region and the overall effect of stimulation results from the interactions among these cells (*38*). Therefore, it is unclear how cortical neuromodulation contributes to enhancing post-stroke recovery. In this sense, ChR2 and opsin-based optogenetic tools have gained considerable attention for their ability to spatiotemporally modulate neuronal circuits in diverse physiological and pathological conditions (*39*, *40*). Previous studies have demonstrated that selective modulation of neurons can promote post-stroke recovery through mechanisms associated with synaptic plasticity (*5*, *41*, *42*). Meanwhile, astrocytes differ substantially from neurons because they do not directly produce action potentials. Instead, calcium signals in astrocytes play pivotal roles in various processes, including the release of gliotransmitters such as glutamate, d-serine, GABA, and ATP, ultimately contributing to the intricate regulation of synaptic plasticity. Therefore, selective control of calcium signaling in astrocytes emerges as an attractive option for neuromodulation, obviating the necessity for direct perturbation of neuronal activity. Furthermore, a growing body of evidence underscores the profound and context-dependent impacts of STIM1-mediated calcium signaling in astrocytes on neuronal activity and plasticity (*43*–*45*). In this perspective, OptoSTIM1 stands out as an optimal tool for fine-tuning calcium-mediated astrocyte functions and subsequently modulating neuronal activity. We have previously confirmed the efficacy of OptoSTIM1 variants in robustly elevating calcium concentrations to physiologically relevant levels by activating endogenously expressed calcium-selective channels, thereby initiating associated signaling pathways in astrocytes and in neurons (*22*, *46*, *47*).

ChR2 is another optogenetic tool that can be used to induce calcium signaling through optogenetic stimulation (*23*, *24*). However, there are differences between calcium rise induced by OptoSTIM1 and ChR2 due to their distinct mechanisms of action. ChR2 is a light-gated ion channel that allows the passage of ions, usually cations such as Na^+^ and Ca^2+^ across the cell membrane during optogenetic stimulation. In contrast, OptoSTIM1 is specifically designed to activate calcium-selective CRAC channels located in the plasma membrane. The differential outcomes observed in our study can be attributed to the nonselective cation channel property of ChR2, which may not sufficiently elevate calcium levels to a degree that allows calcium-mediated astrocytic functions to contribute notably to the recovery process. Furthermore, it is noteworthy that while ChR2 regulates intracellular calcium levels on a second timescale, the activation of OptoSTIM1 could induce a prolonged increase in calcium levels for minutes with a higher amplitude, thereby offering an alternative explanation for the observed difference. Consistent with these observations, the activation of ChR2 in astrocytes failed to yield functional recovery of post-stroke disabilities. In contrast, the activation of OptoSTIM1 in astrocytes significantly facilitated the restoration of motor impairments.

Previously, we demonstrated that capsular infarct causes retrograde atrophy in the sensorimotor cortex with tonic inhibition of neighboring neurons by aberrant astrocytic GABA synthesis, leading to the decrease of regional glucose hypometabolism in diaschisis and reduced neuronal activity (*35*, *48*). We also showed that subsequent reduction of diaschisis and reversal of cortical glucose metabolism are closely related to functional recovery following stroke. The cortical sites of stimulation are the critical factor for stroke recovery. Stimulating the cortex is likely to induce different patterns of functional reorganization and activate different neural circuits depending on the stimulation site. Our previous study identified the SPC as the most effective stimulation site (*25*, *49*). Consistent with our previous research, we demonstrated that OptoSTIM1 activation in the SPC led to a significant reduction in cortical diaschisis and restoration of SUVR in that area, both significantly correlated with behavioral outcomes. We also observed that OptoSTIM1 activation increased c-Fos expression not only in the targeted astrocytes but also in nearby neurons. This observation suggests the existence of a neuron-astrocyte interaction mechanism, wherein calcium-dependent release of gliotransmitters, such as glutamate, d-serine, and ATP from astrocytes leads to an increase in neuronal activity and glucose metabolism (*12*, *20*, *23*). Using FDG-microPET imaging, we revealed the restoration of corticocortical circuits following astrocyte calcium modulation. We observed an increase in cortical activity not only in the ipsilesional motor cortex but also in the contralesional motor cortex, indicating interhemispheric interactions. Furthermore, these changes were persistent and did not decline during the observation period. Considering the critical role of corticocortical circuits in integrating information and the coordination of complex behaviors (*50*, *51*), this restoration is likely to contribute to the recovery of motor function after stroke.

At the molecular level, we found a significant increase in the expression of BDNF in both neurons and astrocytes in the bilateral motor cortex following OptoSTIM1 stimulation, consistent with previous studies that reported elevated BDNF expression in the motor cortex during post-stroke recovery (*5*, *52*). The distinct expression of BDNF in neurons and astrocytes suggests their involvement in different cellular processes under stroke conditions. For example, astrocyte-derived BDNF may play a crucial role in modulating neuronal activity and promoting synapse formation (*53*, *54*), while neuron-derived BDNF may contribute to circuit reorganization through axonal and dendritic sprouting (*55*, *56*). Thus, the activation of astrocytes induced by OptoSTIM1 implies multiple BDNF actions involved in post-stroke recovery. Together, we suggest a mechanism where optogenetic modulation of calcium signals in astrocytes leads to a local increase in adjacent neuronal activity, followed by long-range effects on enhancing synaptic activity and plasticity in interconnected motor cortices. This process ultimately results in reorganizing and restoring neuronal circuits during post-stroke recovery.

Management of chronic stroke is crucial due to its long-term implications for health care costs, workforce productivity, caregiver burden, and the quality of life of stroke survivors (*57*, *58*). Despite substantial advances in preventing and managing stroke, neural plasticity to organize and form new neural connection imposes a limited “time window” for functional recovery (*59*). Therefore, a new treatment strategy has to overcome the limits of this time window for post-stroke recovery. In this study, we used a chronic capsular infarct model in rats, as previously described, because this model produces a chronic motor deficit for more than 4 weeks that does not recover naturally (*25*, *60*). Our results demonstrated that OptoSTIM1 astrocytic stimulation, through a daily single session of 1-hour low-intensity light stimulation, significantly improved behavioral recovery with various stroke recovery signals in a chronic stroke model. In addition, our experiments with cultured astrocytes demonstrated that OptoSTIM1 activation neither disrupts basal calcium activity nor causes excessive calcium influx beyond physiological levels. Therefore, this method may be appropriate without causing phototoxicity in the surrounding tissues. In addition, in our experimental setup using a chronic capsular infarct model, rats showed peak behavioral recovery on the 12th day of stimulation, maintained until the end of the observation period. Therefore, we suggest that chronic stimulation for more than 2 weeks is necessary to fully evaluate the effect of stimulation on functional recovery.

In summary, our study provides compelling evidence that selective modulation of astrocyte calcium signals can significantly affect neuronal activity and synaptic plasticity, thereby facilitating post-stroke recovery. On the basis of these findings, we propose that targeting astrocytes could be a promising therapeutic strategy for many neurological disorders, extending beyond stroke recovery. While OptoSTIM1 offers a powerful and precise tool for studying astrocytic calcium signaling in experimental models, translating this approach to clinical applications would require overcoming substantial challenges, including the complexities of delivering and expressing optogenetic modules in human brain tissue, as well as obtaining regulatory approvals for gene therapy–based interventions. Alternatively, pharmacological approaches targeting astrocytic calcium signaling may provide a more feasible pathway for clinical applications. Several compounds that modulate astrocyte-specific calcium channels or relevant neurotransmitter systems could potentially offer a noninvasive and clinically viable means for therapeutic benefit (*61*–*63*). Nonetheless, we anticipate that OptoSTIM1 holds considerable promise in diverse physiological and pathological conditions, allowing for the selective modulation of calcium-mediated functions of astrocytes and other cell types in the brain.

## MATERIALS AND METHODS

### Plasmid construction

Expression plasmids for RGECO1 (Addgene plasmid #32444) and jRGECO1a (Addgene plasmid #61563) were obtained from Addgene. The construction of expression plasmid for OptoSTIM1 was previously described in detail (*22*). For construction of expression plasmid for ChR2(H134R)-mEGFP, the ChR2(H134R) sequence from ChR2-YFP-NavII-III (Addgene plasmid #26057) was amplified by polymerase chain reaction (PCR) using ChR2(H134R)-F (5′-GACTGCTAGCGCCACCATGGACTATGGCGG-3′) and ChR2(H134R)-R (5’-GACTACCGGTGCTGGCACGGCTCCGGC-3′) primers. For construction of virus vector for pLenti-*GfaABC1D*-OptoSTIM1 and pLenti-*GfaABC1D*-EGFP, *GfaABC1D* promoter was amplified by PCR using GfaABC1D-F (5′-GAAAATTAATTAAAGATCTAACATATCCTGGTGTGGAGTAGG-3′) and GfaABC1D-R (5′-GAAAAGGATCCCCGCGAGCAG-3′) primers and inserted into pLenti-*CaMKII*α-OptoSTIM1 and pLenti-*CaMKII*α-EGFP after the excision of *CaMKII*α promoter as described previously (*46*). For construction of virus vector for pLenti-*GfaABC1D*-ChR2-EYFP, ChR2(H134R)-EYFP from pLenti-EF1α-hChR2(H134R)-EYFP (Addgene plasmid #20942) was amplified by PCR using hChR2(H134R)-EYFP-F (5′-CTGCTCGCGGGGATCCATGGACTATGGCGGCG-3′) and hChR2(H134R)-EYFP-R (5′-GCTTGATATCGAATTCTTACTTGTACAGCTCGTCCATGCC-3′) primers and inserted into pLenti-GfaABC1D-OptoSTIM1 after excision of OptoSTIM1.

### Experimental animals

All animal experimental procedures were approved by the Gwangju Institute of Science and Technology Animal Care and Use Committee (GIST-2019-074). Animal ARRIVE guidelines were followed in the preparation of the manuscript. Sixty-three male Sprague-Dawley rats (9 weeks old, ~300 g) were used in this experiment. Animals were housed in a cage with free access to food and water and were kept on a 12-hour light/12-hour dark cycle (7 a.m. to 7 p.m.) at 22°C and 50% humidity.

For the investigation into the effect of astrocytic optogenetic stimulation on capsular stroke, 27 rats underwent Lenti-GfaABC1D-OptoSTIM1 (OptoSTIM1 group, *N* = 9; sham stimulation group, *N* = 9) or Lenti-GfaABC1D-EGFP (control group, *N* = 9) virus injection into SPC and photothrombotic stroke lesioning in the PLIC. In a similar manner, we additionally injected Lenti-GfaABC1D-ChR2-EYFP virus (ChR2 group, *N* = 10), whose stimulation is known to induce astrocytic Ca^2+^ elevation by calcium influx through the nonselective cation channel in the cell membrane. Animals in the OptoSTIM1 and control groups received optogenetic stimulation during the stimulation period, whereas the sham stimulation group did not receive optical stimulation. In addition, to investigate the effect of OptoSTIM1 activation on regional glucose metabolism in nonstroke rats, we injected Lenti-GfaABC1D-OptoSTIM1 (*N* = 8) or Lenti-GfaABC1D-EGFP (*N* = 8) into SPC. For the verification of viral vectors through Ca^2+^ imaging, 10 rats underwent a mixture of AAV-GfaABC1D-jRGECO1a and pLenti-GfaABC1D-OptoSTIM1 (*N* = 5) or Lenti-GfaABC1D-EGFP (*N* = 5) virus injection into SPC.

### Primary astrocyte culture

Primary cortical astrocytes were dissected by removing the adherent meninges from C57BL/6 mouse pups aged postnatal day 0 to 1 (P0–P1) and dissociating the tissue into a single-cell suspension by trituration through a Pasteur pipette. The dissociated cells were then plated on 60-mm dishes coated with poly-d-lysine (50 μg/ml). Cells were maintained in Dulbecco’s modified Eagle’s medium (Gibco) containing high glucose and l-glutamine supplemented with 10% horse serum, 10% fetal bovine serum, and penicillin-streptomycin (1000 U/ml) at 37°C and 5% CO_2_. On the third day of culture, the cells were washed by replacing the medium after removing the floating cells through vigorous pipetting. Cells were transfected using Lipofectamine LTX (Invitrogen) according to the manufacturer’s instructions.

### Live-cell imaging

For imaging, cells were plated on 24-well polymer coverslip-bottom plates (u-plate 24-Well ibiTreat, ibidi). Live-cell imaging was performed using a Nikon A1R confocal microscope equipped with a FI Plan Apochromat VC objective [×60 /1.4–numerical aperture (NA)] and digital zooming of Nikon imaging software (NIS Element AR 64-bit version 3.21; laboratory imaging). Using the Chamlide TC System mounted on the microscope, the cells were maintained at 5% CO_2_ and 37°C during live-cell imaging. For activation of OptoSTIM1 at 1 Hz, blue light (power density: 1 mW mm−^2^) was delivered with a 488-nm laser at 30-s intervals for 2 min. For stimulating ChR2 or OptoSTIM1 at 16 Hz, blue light (power density: 120 mW mm−^2^) was delivered three times with a 488-nm laser at 10-s intervals. To activate endogenous adrenergic receptors, 5 μM norepinephrine (Sigma-Aldrich, St. Louis, USA) was treated.

### Live-cell image processing and analysis

Images were analyzed using Nikon imaging software (NIS-element AR 64-bit version 5.11). Changes in fluorescence intensity of RGECO1 were measured using the “ROI” and “time measurement” tools. A “kymograph” tool was used to draw kymographs.

### Calcium imaging from SPC slices

A mixture of 1 μl of AAV-GfaABC1D-jRGECO1a and 1 μl of Lenti-GfaABC1D-OptoSTIM1 or Lenti-GfaABC1D-EGFP virus was injected into SPC. After 4 weeks of virus expression, the rat was decapitated under isoflurane anesthesia (3%). The brain was quickly removed and placed in an ice-cold oxygenated (95% O_2_ and 5% CO_2_) artificial cerebrospinal fluid [ACSF; 130 mM NaCl, 24 mM NaHCO_3,_ 3.5 mM KCl, 1.25 mM NaH_2_PO_4_,1 mM CaCl_2_, 3 mM MgCl_2_, and 10 mM glucose (pH 7.4)]. The brain region containing SPC was coronally sliced into 300-μm thickness using a vibratome (VT 1000S, Leica Biosystems, IL, USA) and stored in an incubation chamber at room temperature for 1 hour before recording. Slices were transferred to the recording chamber, which was superfused with oxygenated ACSF composed of 130 mM NaCl, 24 mM NaHCO_3_, 3.5 mM KCl, 1.25 mM NaH_2_PO_4_, 1.5 mM CaCl_2_, 1.5 mM MgCl_2_, and 10 mM glucose saturated with 95% O_2_ and 5% CO_2_ at pH 7.4. Images were acquired using a Nikon A1R confocal microscope mounted onto a Nikon Eclipse Ti body and equipped with a 25× water-immersion objective lens (N25X-APO-MP; 1.10 NA, Nikon) and lasers with wavelengths of 488 and 561 nm. Slices were scanned at 5-s intervals for 15 min. After 1-min basal image scanning, light stimulation was performed with a 488-nm laser (6 μW) at 5-s intervals for 5 min. Image analysis was performed with NIS-element (Nikon) and ImageJ software.

### Viral vector injection into the SPC

Rats underwent viral injection into the SPC [anterior/posterior (AP) = −4.0 mm, medio-lateral (ML) = ±3 mm, dorso-ventral (DV) = 1 mm from the bregma]. Animals were anesthetized with a mixture of ketamine hydrochloride (100 mg/kg) and xylazine (7 mg/kg). One microliter of Lenti-GfaABC1D-OptoSTIM1, Lenti-GfaABC1D-ChR2-EYFP, or Lenti-GfaABC1D-EGFP (IBS virus facility, Seoul, Korea) was stereotaxically injected at the target site with a rate of 0.1 μl/min using a 33-gauge NanoFil syringe connected to a micropump (WPI, FL, USA). The needle was then slowly withdrawn after injection. Postoperative pain was controlled with ketoprofen (2 mg/kg, i.m.). For the Ca^2+^ imaging experiment, we injected a mixture of 1 μl of AAV-GfaABC1D-jRGECO1a (IBS virus facility, Seoul, Korea) and 1 μl of Lenti-GfaABC1D-OptoSTIM1 or Lenti-GfaABC1D-EGFP into the SPC using the same procedure.

### Photothrombotic capsular infarction and implantation of optic ferrule

Two weeks after the viral injection, animals underwent photothrombotic stroke lesioning in the PLIC in the contralateral hemisphere to the preferred forelimb. Briefly, animals were anesthetized with a mixture of ketamine hydrochloride and xylazine and placed into a rodent stereotaxic frame. Rectal temperature was maintained at 37.5°C using a heating pad. After a scalp incision, a small hole was made and an optic fiber (core diameter of 62.5 μm and an outer diameter of 125 μm) was stereotaxically inserted into the PLIC (AP = −2.0 mm, ML = ±3.1 mm, DV = 7.8 mm from the bregma). Then, Rose Bengal dye (20 mg/kg) was injected (i.v.) and irradiated for 1.5 min using the green laser (3.7 mW). After laser irradiation, the optical fiber was removed. After induction of stroke lesioning in the PLIC, a custom-made plastic ferrule containing the optical fiber (200-μm core and 230-μm cladding diameter) was stereotaxically implanted in the target site (AP = −4.0 mm, ML = ±3 mm, DV = 0.9 mm from the bregma). The optic ferrule was secured to the skull with C&B Metabond (Parkell, NY, USA). The scalp wound was sutured and treated with ketoprofen (2 mg/kg, i.m.) for postoperative pain control. Animals were given 2 weeks of recovery period from capsular infarct and fiber implantation surgeries.

### Behavioral test

The single-pellet reaching task (SPRT) was used to evaluate skilled motor performance throughout the experimental period. We used a clear Plexiglas (45 cm by 40 cm by 13 cm) box with a 1-cm-wide slit and food shelf in the midline of the frontal wall. Rats were food-restricted to 90% of their body weight to motivate sucrose pellet retrieval (Bio-Serve, Frenchtown, NJ). The preferred handedness of each rat was determined during the pretraining period. A reach was classified as successful if the rat extended a forelimb to grasp the pellet and brought it into the mouth without dropping it. Rats received 20 pellets in a single session during the entire experimental period. The SPRT reaching score was calculated as the percentage of successful reaches.

The open field test was used to measure post-ischemic locomotor activity. Animals were habituated to the open field chamber (50 cm by 50 cm by 45 cm) for 30 min once before the initiation of the experiments. Locomotor activity was measured four times at post-lesion days −16(baseline), −1 (pre-stroke), 14 (post-stroke), and 29 (after 2-week stimulation) (Fig. 1A). During the behavioral sessions, locomotor activity was recorded for 10 min using a video camera placed above the chamber. Parameter analysis included total traveled distance, immobility, and velocity using open-source software (Bonsai; https://open-ephys.org/bonsai).

### Optogenetic stimulation

A laser cable was tethered to the implanted ferrule to deliver 473 nm blue light pulses (20 Hz, 10 ms, 1.5 mW) into the SPC (Changchun New Industries, P.R. China), which was controlled by a functional generator (Master 9, A.M.P.I, Jerusalem, Israel). For the stimulation and control groups, animals received 1 hour of stimulation each day for 2 weeks. The sham group did not receive any light-pulse delivery. During the optogenetic stimulation sessions, animals performed the SPRT test, while the blue light was being delivered.

### MicroPET image acquisition and processing

Longitudinal ^18^F-FDG-PET images were acquired to measure changes in regional glucose metabolism and cortical diaschisis before and after stroke lesioning, as well as optogenetic stimulation. Animals underwent four scanning sessions: The first scan was performed before the stroke lesioning (baseline scan), the second scan was performed 2 weeks after the stroke lesioning (PS −1), and the third and fourth scans were performed 7 and 14 days after optogenetic stimulation (PS 7 and PS 14). Rats were deprived of food for 12 hours before PET image scanning to maintain consistency in blood glucose levels. ^18^F-FDG (0.1 mCi/100 g) was injected via the tail vein under isoflurane anesthesia (1.5%). During a 30-min uptake period, both the stimulation and control groups received optical stimulation. Animals were placed in the microPET scanner (Siemens Medical Solutions, TN, USA) under isoflurane anesthesia (1.5%). A static PET scan was then performed for 25 min, followed by an attenuation-correction computed tomography scan for 5 min. Body temperature, respiration, and heart rate were monitored during the acquisition (BioVet, m2m Imaging Corp., Cleveland, OH, USA). The acquired images were corrected for attenuation and reconstructed with a three-dimensional ordered subset maximization algorithm with maximum a posteriori (3D OSEM/MAP).

Images were processed and analyzed with the MINC toolkit (McConnell Brain Imaging Centre, Montreal Neurological Institute, Montreal, Canada) and the Analysis of Functional NeuroImages (AFNI) packages (National Institutes of Health, MD, USA). All acquired images were spatially normalized to an magnetic resonance imaging template of the Sprague-Dawley rat brain. Then, the voxel intensity of each image was normalized to the mean value of the whole brain. Images were spatially smoothed with an isotropic Gaussian kernel with 1.2-mm full width at half maximum.

For the optogenetic stimulation experiment in nonstroke rats, we performed FDG-microPET scans twice: first as a pre-stimulation scan and then as a post-stimulation scan 3 days later. The scanning procedures and analyses were carried out in the same manner.

### Immunohistochemeistry

After completing the last microPET scanning and behavioral tests, animals were sacrificed and processed for histological evaluations. For c-Fos immunohistochemistry, the OptoSTIM1 and control groups received optical stimulation (473 nm, 20 Hz, 10 ms, 1.5 mW) for 30 min, whereas the sham group did not receive any light delivery. Ninety minutes after optical stimulation, animals were perfused with 0.9% saline, followed by 4% paraformaldehyde (PFA) under ketamine (100 mg/kg body weight) anesthesia. Brains were post-fixed overnight in 4% PFA and cryoprotected in 30% sucrose in phosphate-buffered saline. Then, the brains were serially sectioned with a 40-μm thickness at 200-μm intervals. Hematoxylin and eosin, anti–glial fibrillary acidic protein (GFAP) (1:300; AB5541, Abcam) and anti-neurofilament staining (1:1000; ab8135, Abcam) were performed to confirm the completeness of the PLIC lesion. Images were obtained using an Olympus VS200 slide scanner (SLIDEVIEW VS200, Olympus, Tokyo, Japan) with a 20× air objective lens. Infarct volume was measured using ImageJ.

Fluorescent immunohistochemistry was performed on the brain sections. Anti-GFP (1:1000; ab1218, Abcam) and 4′,6-diamidino-2-phenylindole staining were used to confirm viral expression. Anti-BDNF staining (1:500; ANT-010, Alomone) was performed to detect the activity-dependent neurotrophic factor. c-Fos staining (1:1000; 2250S, Cell Signaling Technology) was used as an activity marker. Neuronal nuclei staining was performed with anti-NeuN (1:1000; ABN90P, Millipore). Fluorescent secondary antibodies were purchased from Invitrogen or Jackson ImmunoResearch and used in 1:200 dilutions. Fluorescence images (1024 × 1024 or 2048 × 2048) were obtained using an Olympus FV3000 confocal laser scanning microscope (FLUOVIEW FV3000, Olympus, Tokyo, Japan) and a 20× objective lens. To quantify c-Fos^+^ cells, we selected ROIs from the OptoSTIM1-expressing or EGFP-expressing area. Then, we counted the number of c-Fos^+^ cells colocalized with NeuN^+^ or surrounded by EGFP signals. To quantify BDNF intensity in NeuN^+^ and GFAP^+^ cells, images were converted to an 8-bit image, and NeuN^+^ and GFAP^+^ images were converted into binary images. The binary images and BDNF^+^ images were multiplied so that the BDNF signal remained only. Then, we measured the mean intensity value of BDNF in each ROI. We normalized the BDNF signal intensities of the sham and optoSTIM1 groups to that of the control group. Image analysis was performed using ImageJ.

### Statistical analysis

FDG-microPET images were statistically analyzed with a group-level linear mixed-effect model (3dLME in AFNI). Image analysis compared pre-lesional (baseline) and post-lesional images (PS −1, PS 7, and PS 14) to identify longitudinal changes in cortical diaschisis and regional glucose metabolism after astrocytic optogenetic stimulation. Statistical maps were corrected and thresholded at a significance level (*P* < 0.001, FDR of *q* < 0.05). In addition, image analysis was performed to assess the effects of astrocytic optogenetic stimulation by comparing the pre-stimulation image (PS −1) with post-stimulation images (PS 7 and PS 14). Statistical maps were thresholded at a significance level (*P* < 0.01) and corrected for multiple comparisons with 3dClustSim in AFNI (α = 0.05, *P* < 0.01, *k* < 39). Furthermore, to investigate the changes in glucose metabolism induced by OptoSTIM1 activation in nonstroke rats, we conducted a voxel-wise paired *t* test with 3dttest in AFNI to compare pre-stimulation versus post-stimulation scans (3dClustSim, α = 0.05, *P* < 0.01, *k* < 39). The statistical maps were overlaid on the template to show areas of significant brain activity changes. To quantify the longitudinal metabolic changes in a specific brain region, we used SUVR, as it reduces the influence of variations in injected radio-isotope dose, body weight, or scanner calibration across different time points. SUVR was calculated by dividing the SUV of the ROI by the mean SUV of the entire brain. To measure the longitudinal changes in SUVR of the cortical diaschisis area, ROI masks were created on the basis of statistically thresholded maps of PS −1 for each group. ROIs were defined manually in bilateral motor and ipsilesional sensory cortex to measure the longitudinal metabolic changes.

Statistical analyses were performed using Prism 9 (GraphPad, San Diego, CA, USA). The infarct volume and immunostaining data were analyzed using one-way analysis of variance (ANOVA) with Tukey’s multiple comparisons (*P* < 0.05). Peak calcium signal data were analyzed using repeated-measures two-way ANOVA with Sidak’s multiple comparisons (*P* < 0.05). Changes in daily SPRT performance, cortical diaschisis volume, and SUVR data were analyzed using repeated-measures two-way ANOVA with Geisser-Greenhouse correction and Tukey’s multiple comparisons (*P* < 0.05). Changes in SPRT performance before and after optogenetic stimulation were analyzed using repeated-measures two-way ANOVA with Sidak’s multiple comparisons. The open field test data were analyzed using repeated-measures two-way ANOVA with Tukey’s or Sidak’s multiple comparisons. In addition, linear regression was performed between the SUVR data and SPRT scores and between cortical diaschisis data and SPRT scores using Pearson’s correlation (*P* < 0.05). All data are represented as means ± SEM.
