# Supplementary material for: Optogenetic calcium modulation in astrocytes enhances post-stroke recovery in chronic capsular infarct
Source: Sci Adv. 2025 Jan 31;11(5):eadn7577. doi: 10.1126/sciadv.adn7577 (PMC11784845; doi:10.1126/sciadv.adn7577)
Supplement: Supplementary file 1 — Figs. S1 to S8 Legends for movies S1 to S3 Legend for data S1 [file sciadv.adn7577_sm.pdf]

Science Advances
AAAS

Supplementary Materials for

Optogenetic calcium modulation in astrocytes enhances post-stroke recovery in chronic capsular infarct

Jongwook Cho *et al.*

Corresponding author: Won Do Heo, wondo@kaist.ac.kr; C. Justin Lee, cjl@ibs.re.kr; Hyoung-Ihl Kim, hyoungihl@gist.ac.kr

Sci. Adv. **11**, eadn7577 (2025)
DOI: 10.1126/sciadv.adn7577

The PDF file includes:

Figs. S1 to S8
Legends for movies S1 to S3
Legend for data S1

Other Supplementary Material for this manuscript includes the following:

Movies S1 to S3
Data S1

Supplementary figures

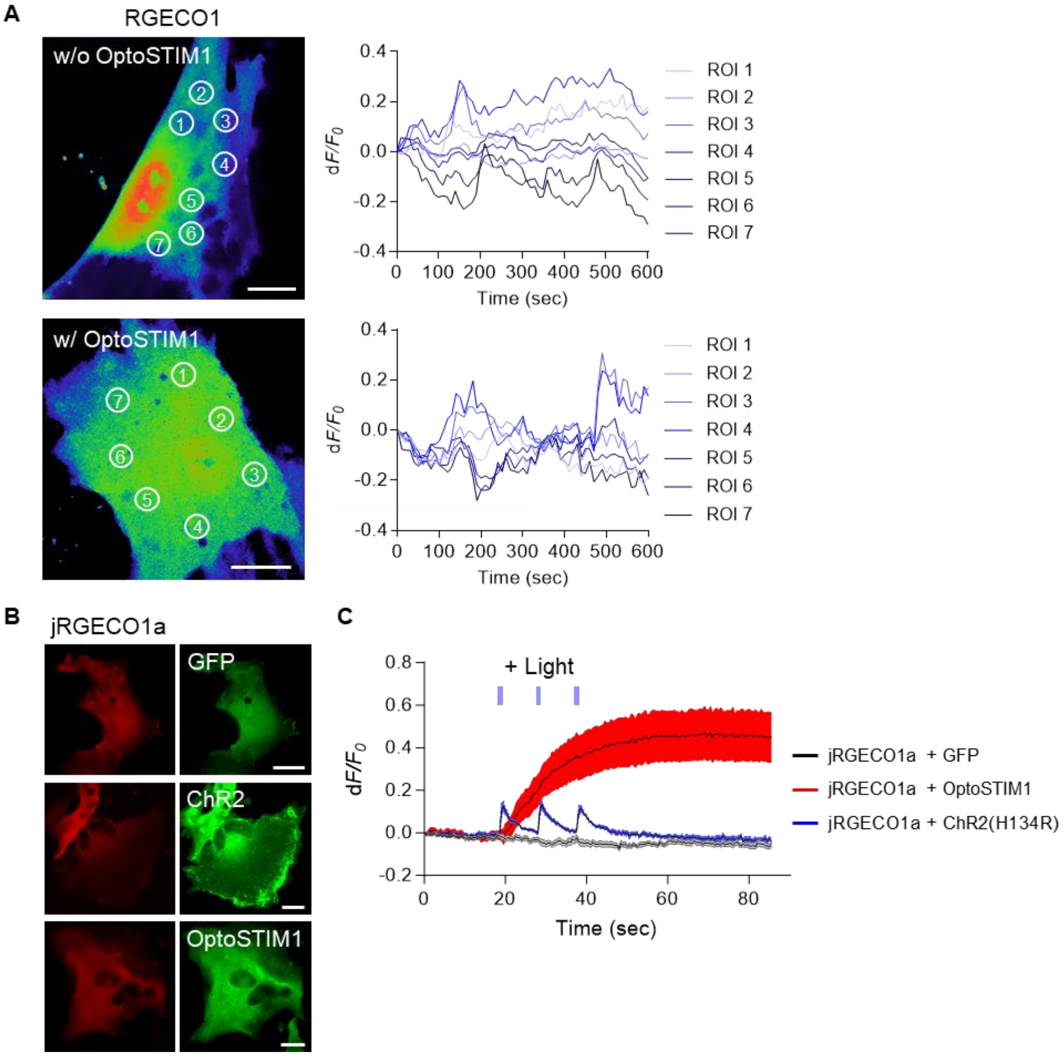

Fig. S1. Effect of OptoSTIM1 on basal calcium activity in the dark and calcium influx under light stimulation.

(A) (Left) Fluorescence images of cultured astrocytes expressing RGECO1 in the absence or presence of OptoSTIM1. Scale bars, 20 µm. (Right) Graphs illustrating changes in RGECO1 intensity over time in different subcellular regions (ROIs 1-7), indicated by white circles in the left images. **(B)** Fluorescence images of cultured astrocytes expressing jRGECO1a along with GFP, OptoSTIM1 or ChR2(H134R). Scale bars, 20 µm. **(C)** A graph illustrating changes in jRGECO1a intensity upon light illumination at 16 Hz.

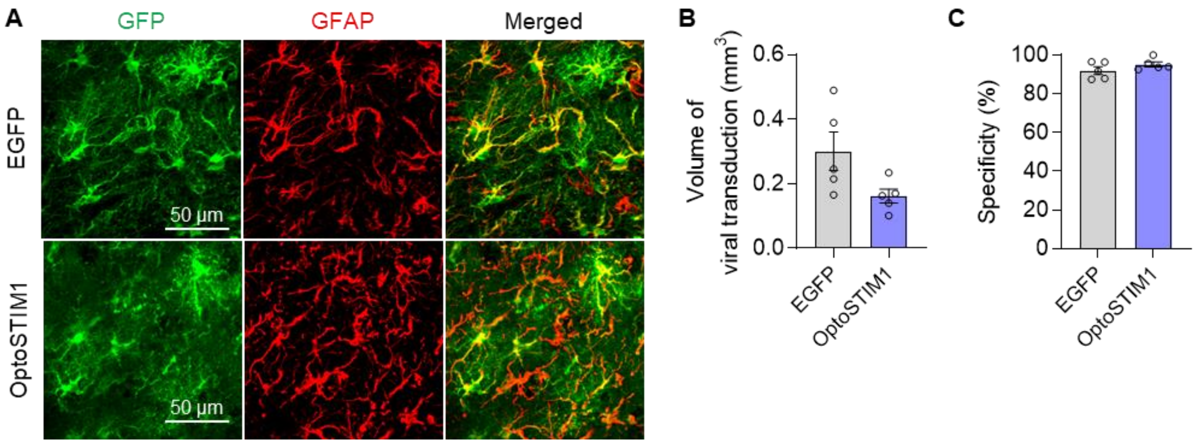

Fig. S2. Viral specificity of Lenti-GfaABC1D-EGFP and Lenti-GfaABC1D-OptoSTIM1.
(A) Representative confocal images showing the co-expression of GFP^{+} and $GFAP^{+}$ cells in the sensory-parietal cortex. **(B)** Volume of viral transduction (unpaired t-test, t = 2.183, p = 0.0605). **(C)** Mean proportion of co-localization of GFP^{+} and $GFAP^{+}$ cells in virus-expressing areas (unpaired t-test, t = 1.348, p = 0.2146). Error bars represent mean ± SEM.

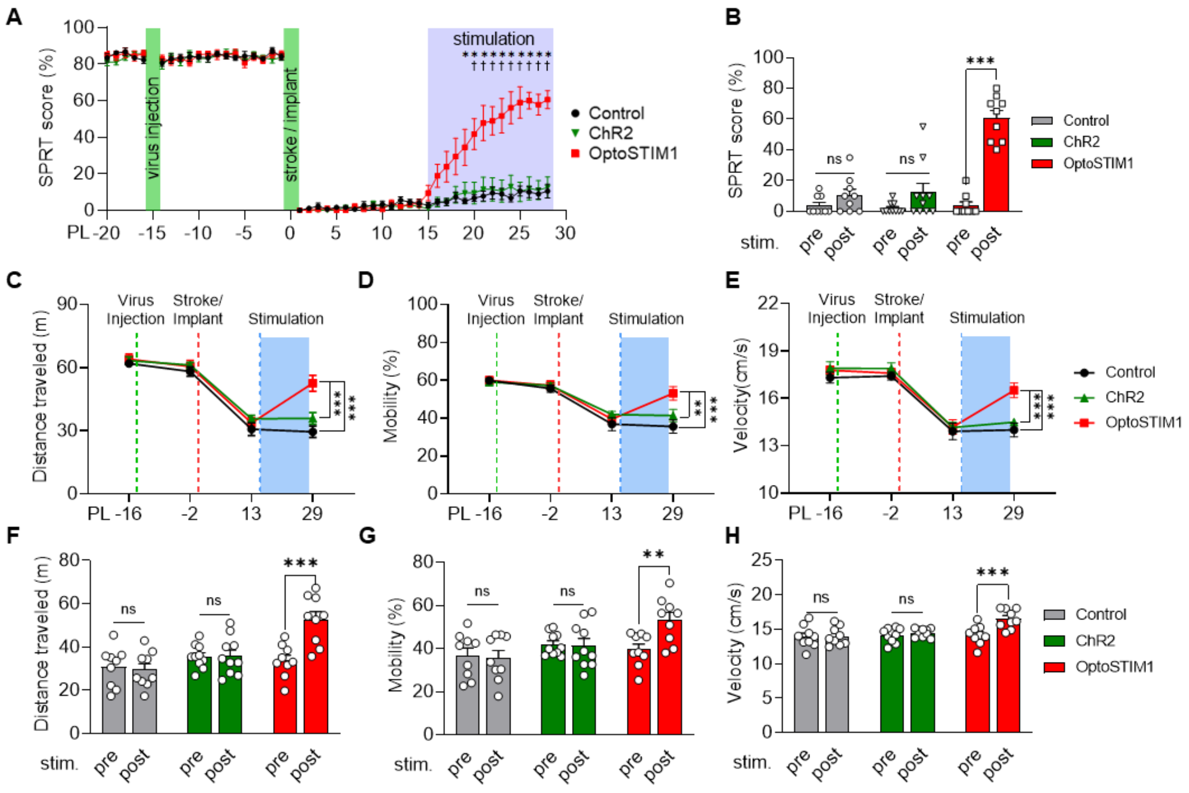

Fig. S3. ChR2 activation in astrocytes of the sensory-parietal cortex did not improve motor deficits in a chronic stroke model.

(A) Daily performance of SPRT in the control, ChR2, and OptoSTIM1 groups. (Repeated-measures two-way ANOVA with Geisser-Greenhouse correction and Tukey's multiple comparisons, F(92, 1150) = 11.33, $p < 0.0001$; *OptoSTIM1 vs. Control; †OptoSTIM1 vs. ChR2). The purple-shaded area indicates the period of optical stimulation. **(B)** Changes in SPRT performance after optogenetic stimulation (Repeated-measures two-way ANOVA with Sidak's multiple comparisons, F(2, 25) = 39.94, $p < 0.0001$). **(C-E)** Changes in locomotor activity in the open field arena, showing distance traveled (m), mobility (%), and locomotor velocity (cm/s) (Repeated-measures two-way ANOVA with Tukey's multiple comparisons, C, F(6, 75) = 5.393, $p = 0.0001$; D, F(6, 75) = 2.992, $p = 0.0113$; E, F(6, 75) = 3.054, $p = 0.010$). **(F-H)** Changes in distance traveled (m), mobility (%), and locomotor velocity (cm/s) before and after optogenetic stimulation (Repeated-measures two-way ANOVA with Sidak's multiple comparisons, F, F(2, 25) = 9.056, $p = 0.0011$; G, F(2, 25) = 4.392, $p = 0.0232$; H, F(2, 25) = 7.371, p = 0.0030). Error bars represent mean ± SEM. **$p < 0.01$, ***$p < 0.001$, ns, non-significant.

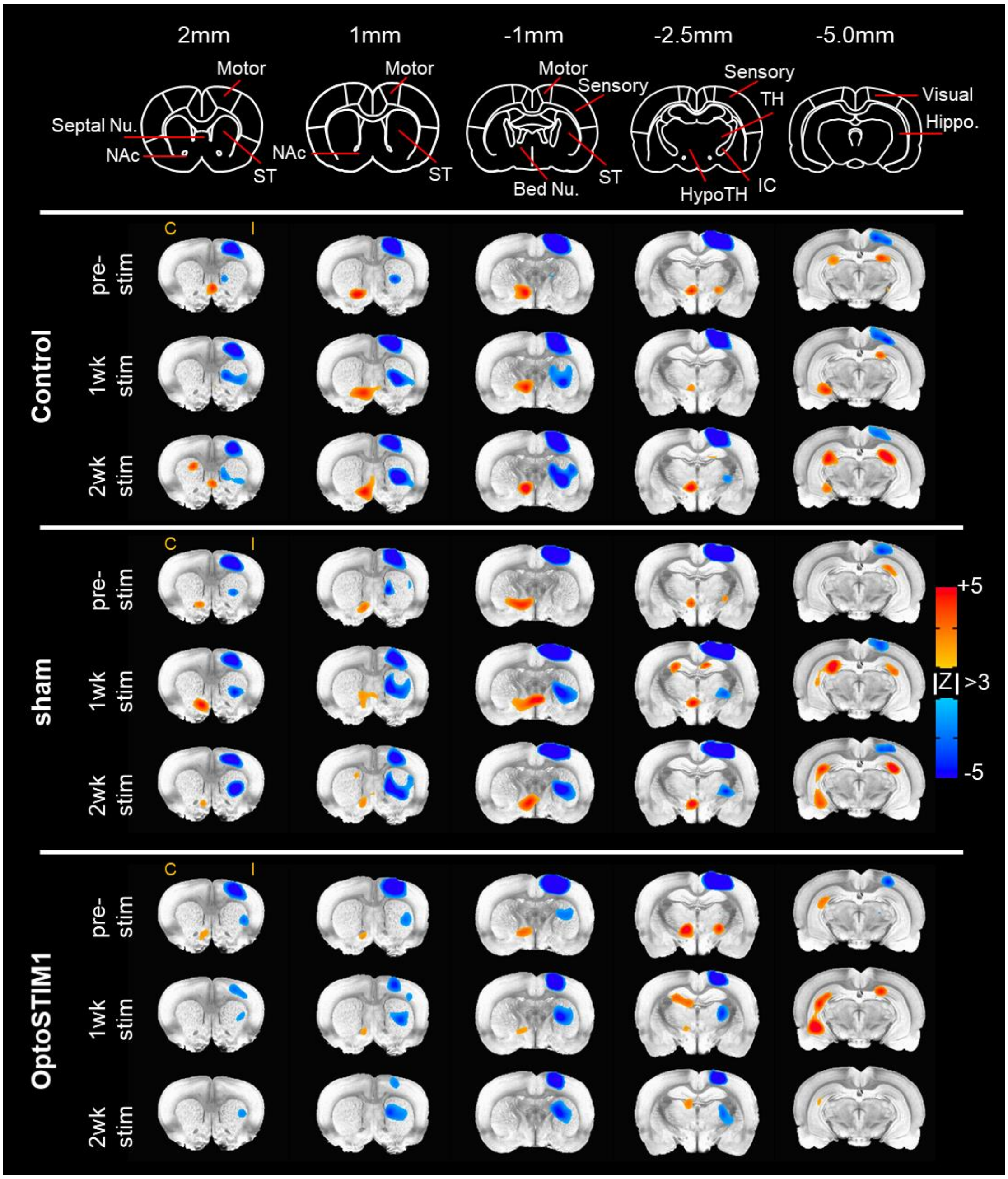

Fig. S4. Longitudinal changes in regional glucose metabolism after optogenetic stimulation in the sensory-parietal cortex.

Color-coded maps showing the activated and deactivated regions in three different groups (3dLME in AFNI, $p = 0.001$, false discovery rate $q < 0.05$). The color scale bar represents z-scores, where positive values (orange-red) indicate regions with increased glucose metabolism relative to baseline, and negative values (blue) indicate regions with decreased glucose metabolism. Septal Nu., septal nucleus; NAc, nucleus accumbens; ST, striatum; Bed Nu., bed nucleus; IC, internal capsule; TH, thalamus; HypoTH, hypothalamus; Hippo, hippocampus; C, contralesional; I, ipsilesional.

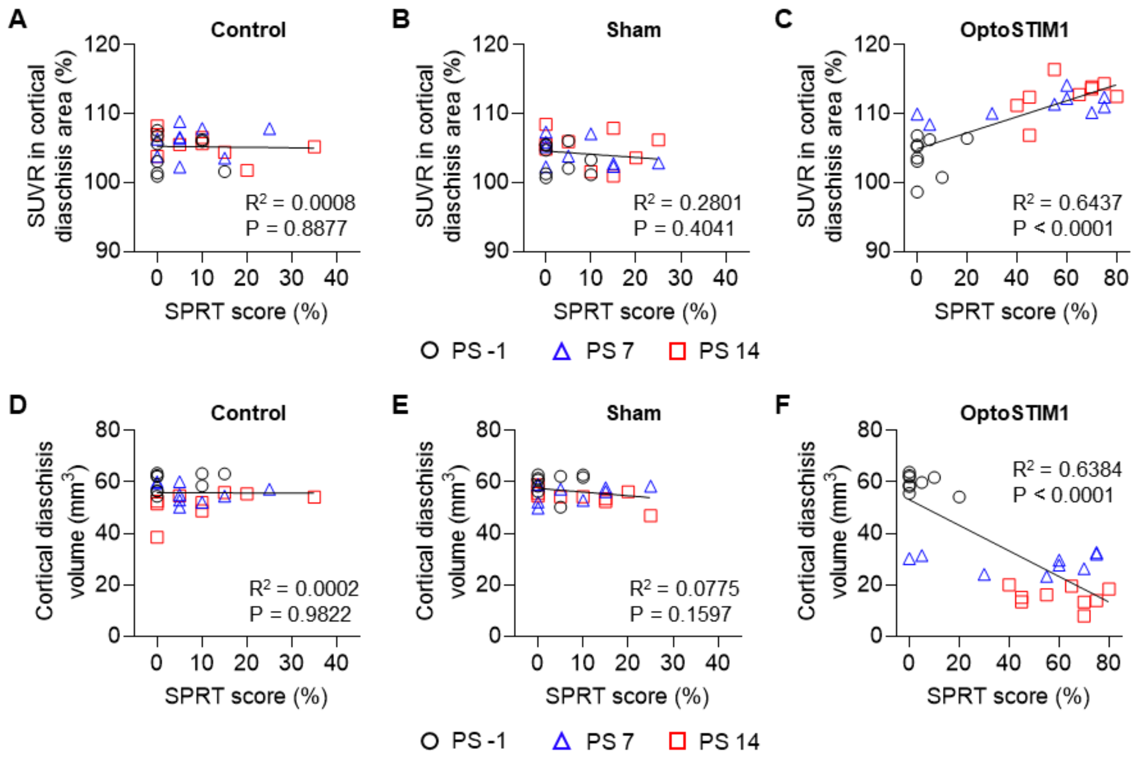

Fig. S5. Reversal of cortical diaschisis significantly correlated with functional recovery. **(A-C)** Change in SUVR in the cortical diaschisis area was positively correlated with SPRT performance in the OptoSTIM1 group (Linear regression, A, $F(1, 25) = 0.02034$, $p = 0.8877$; B, $F(1, 25) = 0.7203$, $p = 0.4041$; C, $F(1, 25) = 45.17$, $p < 0.0001$). **(D-F)** Change in cortical diaschisis volume was negatively correlated with SPRT performance in the OptoSTIM1 group (Linear regression, D, $F(1, 25) = 0.0005$, $p = 0.9822$; E, $F(1, 25) = 2.101$, $p = 0.1597$; F, $F(1, 25) = 44.13$, $p < 0.0001$).

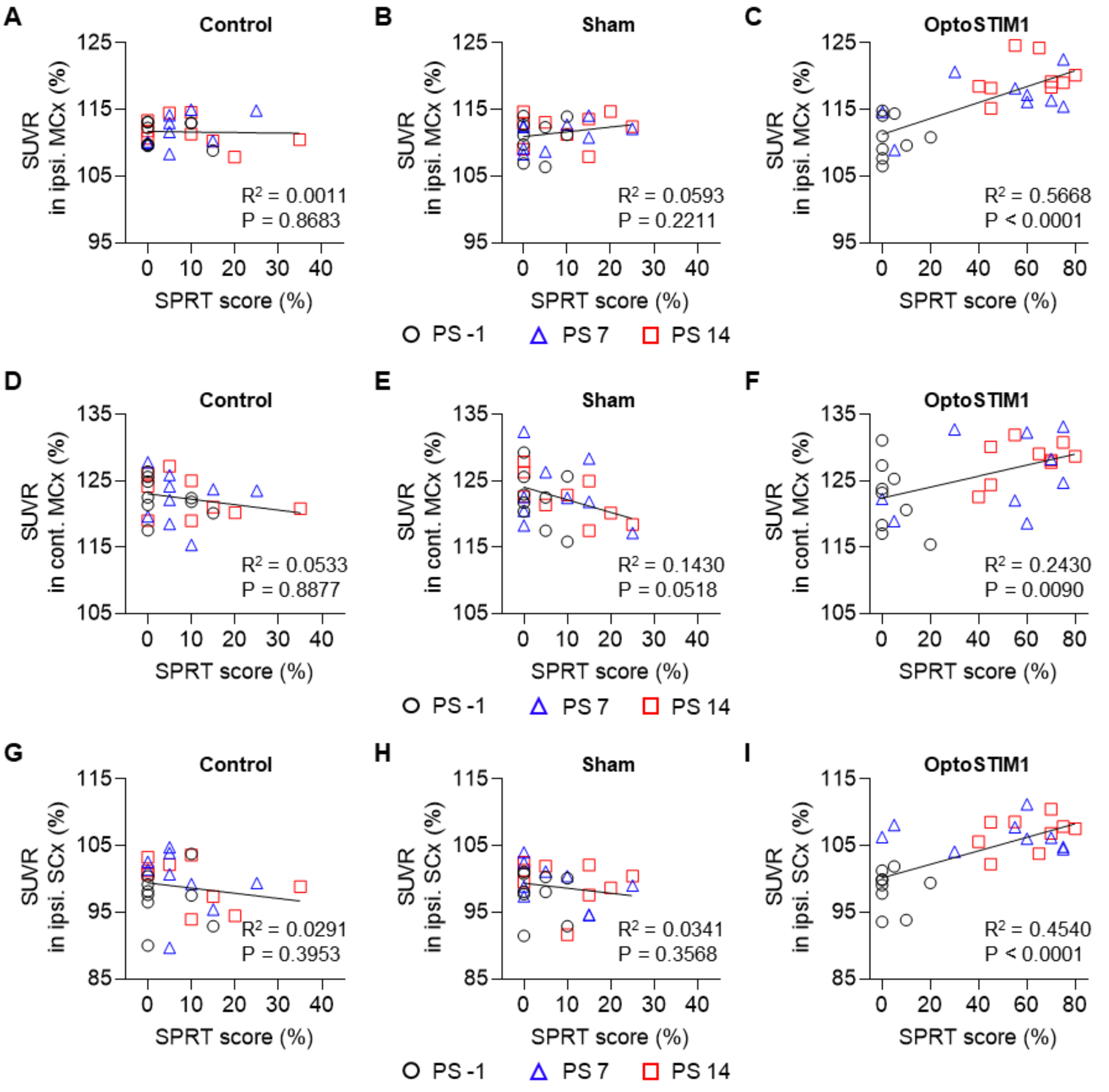

Fig. S6. Involvement of corticocortical circuits following optogenetic astrocytic stimulation after stroke.

(A-C) Change in SUVR in the ipsilateral motor cortex was positively correlated with SPRT performance in the OptoSTIM1 group (Linear regression, A, F(1, 25) = 0.02807, p = 0.8683; B, F(1, 25) = 1.575, p = 0.2211; C, F(1, 25) = 32.71, $p < 0.0001$). **(D-F)** Change in SUVR in the contralateral motor cortex was positively correlated with SPRT performance in the OptoSTIM1 group (Linear regression, D, F(1, 25) = 1.408, p = 0.2465; E, F(1, 25) = 4.170, p = 0.0518; F, F(1, 25) = 8.025, p = 0.0090). **(G-I)** Change in SUVR in the ipsilateral sensory cortex was positively correlated with SPRT performance in the OptoSTIM1 group (Linear regression, G, F(1, 25) = 0.7480, p = 0.3953; H, F(1, 25) = 0.8816, p = 0.3568; I, F(1, 25) = 20.79, $p < 0.0001$).

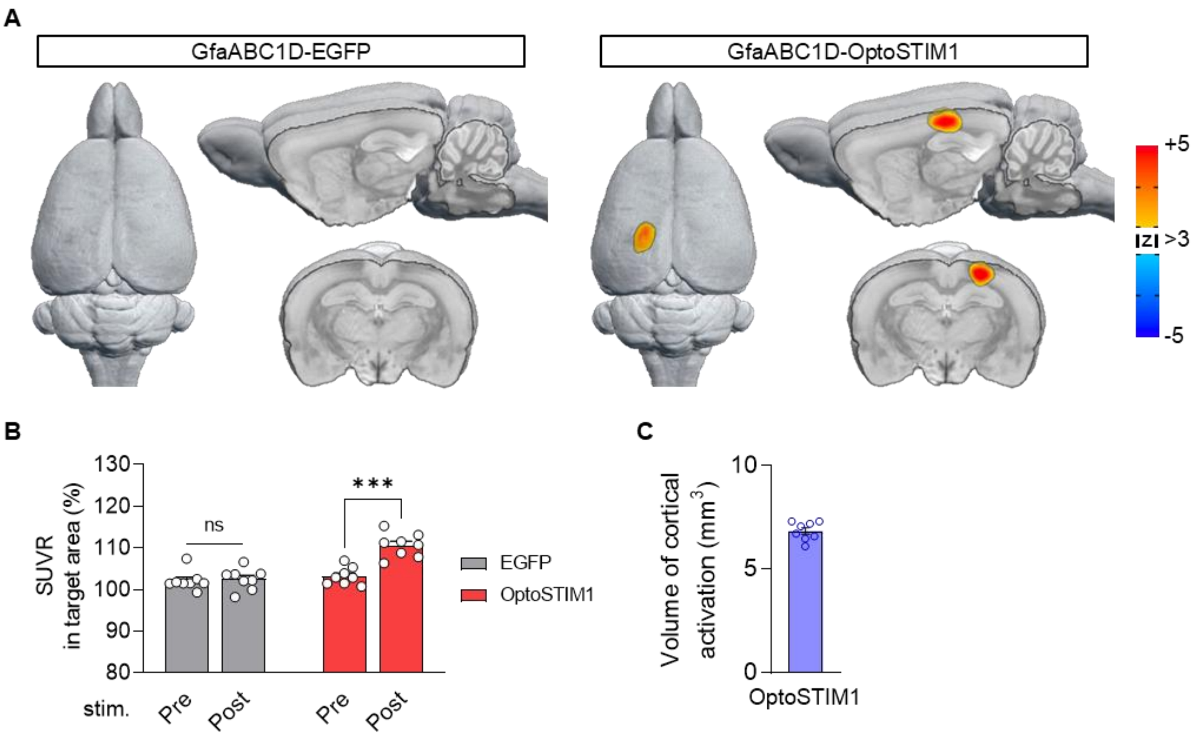

Fig. S7. Change in regional glucose metabolism induced by OptoSTIM1 activation in naive rats.

(A) 3D-rendered FDG-microPET images showing activation of the target area following optogenetic astrocytic stimulation with OptoSTIM1 in non-stroke rats (3dClustSim in AFNI, $p = 0.01$, $\alpha = 0.05$, $k < 39$). The color scale bar represents z-scores, where positive values (orange-red) indicate regions with increased glucose metabolism relative to baseline. **(B)** Change in SUVR in the target area following OptoSTIM1 astrocytic stimulation (Repeated-measures two-way ANOVA with Sidak's multiple comparisons, $F(1, 14) = 38.65$, $p < 0.0001$). **(C)** Volume of cortical activation in the OptoSTIM1 group. Error bars represent mean ± SEM. ***$p < 0.001$, ns, non-significant.

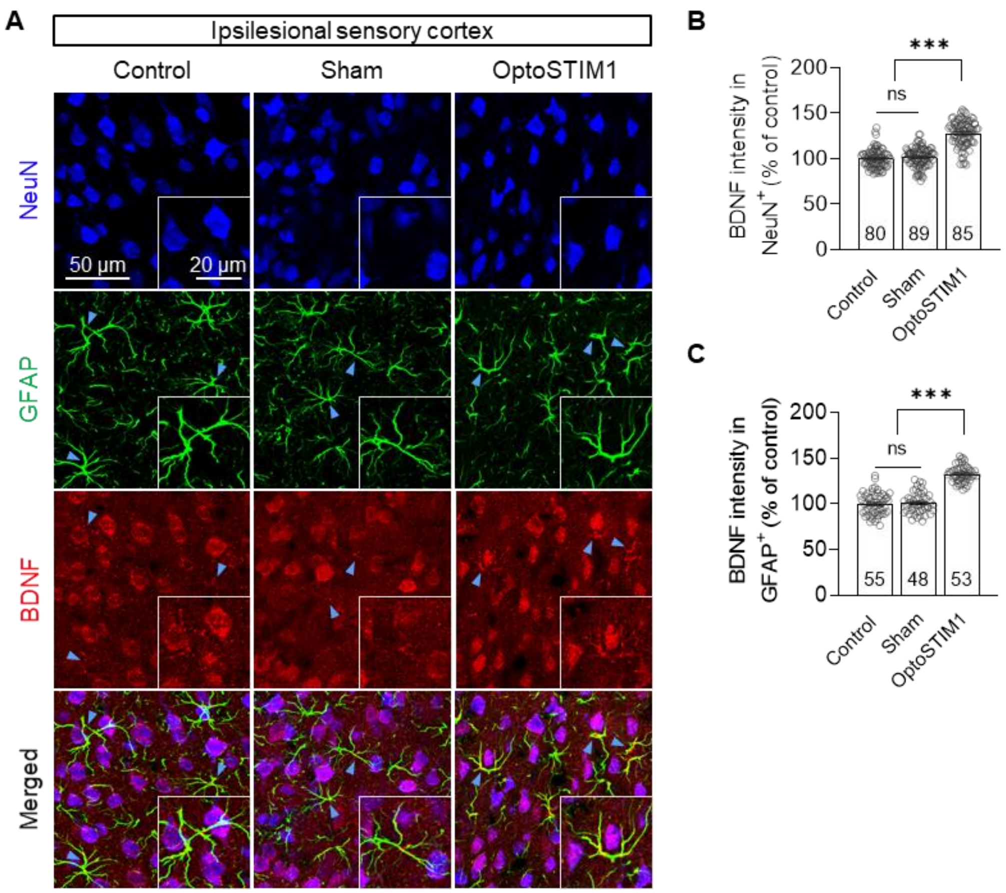

Fig. S8. Astrocytic calcium modulation using OptoSTIM1 enhances BDNF expression after chronic capsular stroke.

(A) Representative confocal images showing staining of NeuN, GFAP, and BDNF in the ipsilesional sensory cortex. **(B)** Quantification of BDNF intensity in NeuN^{+} neurons in the ipsilesional sensory cortex (one-way ANOVA with Tukey's multiple comparisons, $F(2, 251) = 146.8$, $p < 0.0001$). **(C)** Quantification of BDNF intensity in GFAP^{+} astrocytes in the ipsilesional sensory cortex (one-way ANOVA with Tukey's multiple comparisons, $F(2, 153) = 156.7$, $p < 0.0001$). The number on each bar refers to the number of cells analyzed. BDNF-positive astrocytes are indicated by blue arrows. Error bars represent mean ± SEM. ***$p < 0.001$, ns, non-significant.

Movie S1. Basal calcium activity in cultured astrocytes with or without OptoSTIM1 expression.
Cultured astrocytes were transfected with RGECO1, either without (left) or with (right) OptoSTIM1 expression. After a 10-minute of observation period for basal calcium activity, blue light (1 Hz) was administered every 10 seconds for 5 minutes. Fluorescent confocal images of RGECO1 were captured every 10 seconds. The numbers indicate time in minutes:seconds.

Movie S2. Light-induced calcium increase in cultured astrocytes by ChR2 or OptoSTIM1 activation.
Cultured astrocytes were transfected with jRGECO1a, with either ChR2 (left) or OptoSTIM1 (right) expression. Blue light (16 Hz) was administered three times, as indicated. Fluorescent confocal images of jRGECO1a were captured every 500 milliseconds. The numbers indicate time in minutes:seconds.

Movie S3. Light-induced calcium increase in astrocytes in the brain slice by OptoSTIM1 activation.
Astrocytes in the sensory-parietal cortex were transduced with viruses containing jRGECO1a along with either EGFP or OptoSTIM1. Blue light (1 Hz) was administered every 5 seconds for 5 minutes. Two-photon fluorescent images of jRGECO1a were captured every 5 seconds. The numbers indicate time in minutes:seconds.

Data S1. All source data, including the data points from the figures in this manuscript.
